# Effects of Maternal Fructose Intake on Perinatal ER-Stress: A Defective XBP1s Nuclear Translocation Affects the ER-stress Resolution

**DOI:** 10.3390/nu11081935

**Published:** 2019-08-17

**Authors:** Silvia Rodrigo, María I. Panadero, Elena Fauste, Lourdes Rodríguez, Núria Roglans, Juan J. Álvarez-Millán, Paola Otero, Juan C. Laguna, Carlos Bocos

**Affiliations:** 1 Facultad de Farmacia, Universidad San Pablo-CEU, CEU Universities, Montepríncipe, Boadilla del Monte, 28668 Madrid, Spain; 2 Facultad de Farmacia, Universidad de Barcelona, CIBERobn, IBUB, Avda. Joan XXIII 27-31, 08028 Barcelona, Spain; 3 CQS Lab, Calle Marie Curie 5, Rivas-Vaciamadrid, 28521 Madrid, Spain

**Keywords:** fructose, pregnancy, ER stress, methylglyoxal, XBP1s

## Abstract

Endoplasmic reticulum (ER) homeostasis is crucial to appropriate cell functioning, and when disturbed, a safeguard system called unfolded protein response (UPR) is activated. Fructose consumption modifies ER homeostasis and has been related to metabolic syndrome. However, fructose sweetened beverages intake is allowed during gestation. Therefore, we investigate whether maternal fructose intake affects the ER status and induces UPR. Thus, administrating liquid fructose (10% w/v) to pregnant rats partially activated the ER-stress in maternal and fetal liver and placenta. In fact, a fructose-induced increase in the levels of pIRE1 (phosphorylated inositol requiring enzyme-1) and its downstream effector, X-box binding protein-1 spliced form (XBP1s), was observed. XBP1s is a key transcription factor, however, XBP1s nuclear translocation and the expression of its target genes were reduced in the liver of the carbohydrate-fed mothers, and specifically diminished in the fetal liver and placenta in the fructose-fed mothers. These XBP1s target genes belong to the ER-associated protein degradation (ERAD) system, used to buffer ER-stress and to restore ER-homeostasis. It is known that XBP1s needs to form a complex with diverse proteins to migrate into the nucleus. Since methylglyoxal (MGO) content, a precursor of advanced glycation endproducts (AGE), was augmented in the three tissues in the fructose-fed mothers and has been related to interfere with the functioning of many proteins, the role of MGO in XBP1s migration should not be discarded. In conclusion, maternal fructose intake produces ER-stress, but without XBP1s nuclear migration. Therefore, a complete activation of UPR that would resolve ER-stress is lacking. A state of fructose-induced oxidative stress is probably involved.

## 1. Introduction

In the last few decades, the prevalence of diseases related to metabolic syndrome has increased worldwide. This is why, in 2002, the World Health Organization declared obesity as an epidemic disease in the 21st century. Moreover, in 2011, the United Nations declared for the first time that non-communicable diseases such as diabetes, cardiovascular events, or cancer, presented a higher prevalence than infectious diseases around the world [1]. The emergence of these disorders has been mainly promoted by the introduction of unhealthy nutritional habits and a sedentary life style [2]. These habits include the consumption of a wide variety of processed foods and sugary drinks with added sugars [3], such as fructose.

Fructose is a monosaccharide naturally present in fruits, vegetables, and honey. Although its use as a sweetener is relatively recent, since the 1970s it has become a protagonist in the Western diet. Its use has soared dramatically, gaining ever-increasing presence in the formulation of various foods such as cereals, precooked food, jams, juices but, especially, in soft drinks and sweetened beverages [4]. Fructose has been linked to the onset of the metabolic syndrome-related disturbances such as insulin resistance, high blood pressure [5], and hyperuricemia [6]. These results have not only been detected in animal models, but several clinical studies also point to fructose as a cause of metabolic syndrome [7,8].

On the other hand, the stress of endoplasmic reticulum (ER) has recently been related to the development of diseases associated with metabolic syndrome. In particular, ER-stress has been shown to be a central mechanism involved in the onset of atherosclerosis, fatty liver disease, obesity, insulin resistance and type 2 diabetes [9,10,11]. Among the effects related to ER-stress are: the expression and accumulation of mutant or misfolded proteins, excessive lipid accretion, and abnormalities in the intracellular flows of energy [12].

The appearance of ER-stress during pregnancy has also been studied, but in less depth. Recent investigations have found a relationship between an increased ER-stress and placental oxidative stress with the early onset of pre-eclampsia or intrauterine growth retardation [13]. Moreover, there are several articles which hypothesize the possibility that the development of ER-stress during pregnancy can produce a fetal programming in such a way that it will lead to the development of insulin resistance in the offspring at the postnatal stage [14].

When ER homeostasis is disturbed, the unfolded protein response (UPR) is activated. This UPR seeks to mitigate the adverse effects of ER-stress, by decreasing the translation of mRNA and thus reducing the arrival of proteins to the ER to be folded, facilitating the degradation of incorrectly folded proteins and increasing the production of chaperones involved in protein folding [15,16]. The UPR displays three branches: IRE1 (inositol requiring enzyme 1), PERK (protein kinase RNA-like endoplasmic reticulum kinase), and ATF6 (activating transcription factor 6) [15,16].

Regarding the effects of fructose consumption on ER-stress, there is some controversy. Whereas some reports have reported no effects, other studies support fructose as a cause of ER-stress [17,18,19]. In fact, some of the authors pointed out the length of the treatment as a critical factor to explain the differences found in the UPR and ER-stress between studies [19].

Several mechanisms have been proposed trying to explain how fructose affects cellular ER-stress: (1) Fructose diminishes peroxisome proliferator-activated receptor alpha (PPARα) expression and activity [20] and a defective PPARα signaling has been linked to an induced ER stress [21]. (2) A short-term administration of fructose to male rats induces changes in many oxidative stress markers as well as in the antioxidant system [22]. The accumulation of misfolded proteins in the endoplasmic reticulum is enhanced under conditions of oxidative stress and results in ER stress. In fact, antioxidants suppress oxidative stress directly and also the subsequent ER stress [23]. (3) Besides reactive oxygen species, other cellular mediators of fructose-induced hepatic stress signaling have been proposed: carbohydrate intermediates, such as phosphorylated sugars and lactate; and reactive aldehydes such as methylglyoxal (MGO) [24]. (4) Fructose can activate lipogenesis and perturb membrane properties in ER, leading to UPR [25]. In fact, a crosstalk between SREBP-1c, XBP1s (X-box binding protein 1 spliced form), and lipogenesis has been proposed [26].

It remains to be established whether maternal fructose intake provokes perinatal ER-stress and the mechanisms involved in this process. In fact, only Clayton et al. have investigated the effect of fructose during gestation on ER-stress, using rats who received 20% of fructose as a total caloric intake during pregnancy and until 10 days after parturition [27]. Regarding the possible mechanisms involved in ER-stress, we have previously reported in our animal model of fructose ingestion during gestation that, although *PPARα* expression was not affected, a higher expression of genes related to lipogenesis and a lower expression of fatty acid catabolism genes were found in fetuses from fructose-fed mothers [28]. Moreover, we have previously established that maternal fructose induces oxidative stress in fetuses and placentas [29].

Accordingly, the current report is a follow-up study to investigate whether fructose intake (10% wt/vol) throughout gestation is able to produce an increase in ER-stress and affect the UPR in maternal and fetal liver and placenta. Interestingly, our model of maternal liquid fructose intake is confined to the prenatal stage and compares the effects of fructose versus glucose supplementation. Further, in order to elucidate the mechanism of action of fructose-induced changes in ER stress, both the levels of XBP1s, a typical protein of ER stress [17], its translocation to the nucleus and activity as a transcription factor, and the levels of methylglyoxal, were determined in the liver and placenta.

## 2. Materials and Methods

### 2.1. Animals and Experimental Design

Female Sprague-Dawley rats were fed ad libitum standard rat chow (B&K Universal, Barcelona, Spain) and housed under controlled light and temperature conditions (12-h light-dark cycle; 22 ± 1 °C). The experimental protocol was approved by the Animal Research Committee of the University San Pablo-CEU, Madrid, Spain. Rats were mated, and at day 0 of pregnancy, the animals were randomly separated into a control group (*n* = 7), a fructose-fed group (fructose; *n* = 7), and a glucose-supplemented group (glucose; *n* = 6). Fructose and glucose were supplied as a 10% (wt/vol) solution in drinking water throughout gestation. Control animals did not receive any supplementary sugar. Intake of solid food and liquid per cage were recorded daily. The total amount of ingested energy did not differ between fructose-fed, glucose-supplemented, and control rats, as described in [28].

In the morning of the 21st day of pregnancy, rats were decapitated and blood was collected using tubes containing Na2-EDTA. Liver was immediately removed, placed in liquid nitrogen and kept at −80 °C. The conceptus was dissected, placentas obtained and those coming from the same litter pooled and subsequently frozen. Fetuses were decapitated, their livers obtained, and those coming from the same mother pooled (without being separated by gender) and placed in liquid nitrogen to be stored at −80 °C for further analysis.

### 2.2. RNA Preparation and Analysis

Total RNA was isolated from the liver or placenta using Ribopure (Ambion Inc., Austin, TX, USA). RNA was prepared either from the liver of individual animals or from the pools of the same litter for fetal livers or placentas. Total RNA was subjected to DNase I treatment using Turbo DNA-free (Ambion Inc.), and RNA integrity was confirmed by agarose gel electrophoresis. Afterward, cDNA was synthesized by oligo(dT)-primed reverse transcription with Superscript II (Invitrogen, Carlsbad, CA, USA). qPCRs were carried out using a Light Cycler 1.5 (Roche, Mannheim, Germany). The reaction solution was performed in a volume of 20 μL, containing 10 pmol of both forward and reverse primers, 10× SYBR Premix Ex Taq (Takara Bio Inc., Shiga, Japan), and the appropriate nanograms of the cDNA stock. *Rps29* was used as the reference gene for qPCR [28]. The primer sequences for target genes of UPR and XBP1s proteins were obtained either from the Atlas RT-PCR Primer Sequences (Clontech, Palo Alto, CA, USA) or designed using Primer3 software (University of Massachusetts Medical School, Worcester, MA, USA) [30]. Samples were analyzed in duplicate on each assay. Amplification of non-specific targets was discarded using the melting curve analysis method for each amplicon. qPCR efficiency and linearity were assessed by optimization of the standard curves for each target. The transcription was quantified with Light Cycler Software 4.05 (Roche) using the efficiency correction method [31].

### 2.3. Preparation of Protein Extracts

For total protein extraction, samples were homogenized with a lysis buffer with proteases, phosphatases, and deacetylases inhibitors, and incubated for 1.5 h at 4 °C. Samples were then centrifuged at 15,000× *g* for 15 min at 4 °C and the supernatants collected. To obtain hepatic nuclear extracts, samples were homogenized with a homogenization buffer, kept on ice and centrifuged at 1000× *g* for 10 min at 4 °C. Lysis buffer was added to the pellet that was obtained and samples were incubated for 1.5 h at 4 °C, centrifuged at 25,000× *g* for 30 min at 4 °C, and the supernatants collected. The composition of the buffers was as described previously [32].

### 2.4. Western Blot Analysis

Thirty micrograms of different protein fractions from rat tissues were subjected to 10% SDS-polyacrylamide gel electrophoresis. Proteins were transferred to Immobilon polyvinylidene difluoride transfer membranes (Millipore, Burlington, MA, USA) and blocked for 1 h at room temperature with 5% non-fat milk solution in 0.1% Tween-20-Tris-buffered saline (TBS). Membranes were then incubated overnight with the primary antibody in 0.1% Tween-20-TBS with 5% bovine serum albumin (BSA) at 4 °C. Detection was achieved using the enhanced chemiluminescence (ECL) kit for horseradish peroxidase (HRP) (Amersham GE Healthcare Europe GmbH, Barcelona, Spain). To confirm the uniformity of protein loading, the blots were incubated with β-actin or β-tubulin antibody (Sigma-Aldrich, Saint Louis, MO, USA) as a control. The size of the detected proteins was estimated using protein molecular-mass standards (Invitrogen, Life Technologies). Primary antibodies for phosphor- and total-IRE1, ATF6α, phosphor- and total-PERK, and XBP1s proteins were obtained from Santa Cruz Biotechnologies (Dallas, TX, USA) and AbCam (Cambridge, UK) as described previously [32].

### 2.5. Other Determinations

One hundred milligrams of frozen liver (or placenta) were homogenized into PBS buffer and centrifuged (22.000× *g*, 10 min, 5 °C) to remove the precipitate. The concentration of methylglyoxal (MGO) was measured as a precursor of advanced glycation endproducts (AGE) using the method previously described [33] with modifications, by derivatization with *o*-phenylenediamine (o-PD) resulting 2-methylquinoxaline (2-MQ). Perchloric acid (50 μL, 10 mM) and 5-methylquinoxaline (as internal standard; 50 μL, 10 μM) were added to 100 μL of the tissue homogenate solution. Samples were incubated overnight at 5 °C in the dark. Samples (200 μL) were then loaded into a prepared Captiva ND plate, 0.2 µm, polypropylene (Agilent Techonologies, Santa Clara, CA, USA), and rinsed with 2 mL of acetonitrile. Eluted samples were then filtered through 0.2 μm filters (Merck Ltd., Tokyo, Japan) into sample vials. Quantitative analysis of 2-methylquinoxaline was made by triple quadrupole LC/MS (liquid chromatograph/mass spectrometer) (Agilent Technologies 6460, Santa Clara, CA, USA). The MGO concentrations were expressed as nmol/mg of protein.

### 2.6. Statistical Analysis

Results were expressed as means ± S.E. Treatment effects were analyzed by one-way analysis of variance (ANOVA). When treatment effects were significantly different (*P* < 0.05), means were tested by Tukey’s multiple range test, using the computer program SPSS (version 23). When the variance was not homogeneous, a post hoc Tamhane test was performed.

## 3. Results

### 3.1. Ingestion of A 10% w/v Fructose Solution throughout Gestation Partially Activates the UPR Pathway in Liver of Pregnant Rats

Zoometric parameters for these sugar-supplemented rats have been reported previously [28]. Thus, maternal fructose intake during gestation did not change either the body weight of the fetuses or the placental weight by the end of gestation. Neither fructose- nor glucose-fed pregnant rats showed differences between them in glycemia or insulinemia, nor in comparison to control rats. Although total caloric intake was similar in the three groups, the amount of total calories obtained from simple sugars was 25% for the fructose-group and 35% for the glucose-group [28,29]. This amount is similar to the one reported by Clayton et al. in pregnant rats supplemented with fructose [27].

**Figure 1:**
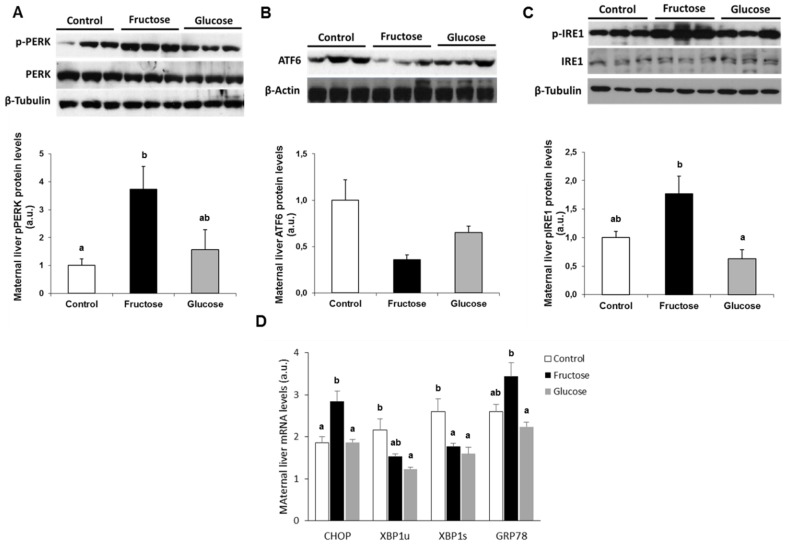
Maternal ingestion of a 10% w/v fructose solution throughout gestation affects maternal ER stress in the liver. Bar plots showing (**A**) the ratio between Thr-phosphorylated form, and the total protein kinase RNA-like endoplasmic reticulum kinase (PERK), (**B**) total activating transcription factor 6 (ATF6), and (**C**) the ratio between Ser-phosphorylated form and the total inositol requiring enzyme 1 (IRE1) proteins in hepatic samples of control (empty bar), fructose (black bar), and glucose-fed (grey bar) pregnant rats. The amount of protein loaded was confirmed by the Bradford method, and the uniformity of protein loading in each lane was assessed by staining the blots with Ponceau S. Values were normalized to β-actin or β-tubulin levels and expressed in arbitrary units (a.u.). (**D**) Bar plots showing the relative hepatic levels of specific mRNA for target genes controlled by the three branches of the unfolded protein response (UPR) from control, fructose, and glucose pregnant rats. Results are the mean ± S.E. of values from 6–7 rats for mRNA expression and 5 rats for protein gene expression. Small letters correspond to the statistical comparisons between rats receiving different supplementation in the drinking water. Values not sharing a common letter are significantly different at *P* < 0.05. CHOP: C/EBP homologous protein; XBP1u: unspliced X-box-binding protein; XBP1s: spliced X-box-binding protein; GRP78: glucose-related protein 78.

In order to study the effect of fructose intake on ER-stress in maternal liver, the three branches of the UPR were analyzed. Figure 1A shows the results obtained on the levels of phosphorylation of PERK (p-PERK) corrected by the protein levels of its total form (PERK) in the liver of the maternal rats. There was an increase in the phosphorylation of this protein in the liver of the mothers supplemented with fructose, this being statistically significant versus the control group. Accordingly, a higher transcript expression of *CHOP*, a downstream effector of PERK [34], was also observed in liver of fructose-fed mothers, and it was statistically different in comparison to both, the glucose-fed and the control groups (Figure 1D).

Unexpectedly, the UPR pathway controlled by ATF6 presented a slight decrease in nuclear levels of this protein for pregnant rats which had received some type of carbohydrate, versus the control group (Figure 1B). It has been described that the induction of ATF6 is concomitant with an increase in gene expression (mRNA) of the transcription factor *XBP1* [35], in the *XBP1u* (X-box binding protein 1 unspliced) form. Therefore, in consonance with the results that we observed for ATF6 protein levels, a decrease in *XBP1u* mRNA levels (Figure 1D) was found in fructose-fed and glucose-supplemented pregnant rats.

Regarding the branch of the UPR controlled by IRE1, Figure 1C shows the results for the phosphorylation of protein IRE (p-IRE1) corrected by the level of its total form (IRE1). As shown in Figure 1C, an increase in the phosphorylation of IRE was observed in the liver of mothers supplemented with fructose, which became significantly different versus the glucose-fed pregnant rats and (marginally) significant (*P* = 0.065) in comparison to the control group. The IRE protein, because of its activity as endoribonuclease, participates in the splicing of the *XBP1u* mRNA to generate the mRNA of *XBP1s* and, therefore, the corresponding protein [35,36]. A decrease in the levels of *XBP1s* mRNA was also observed in the liver of pregnant rats fed with carbohydrates during gestation (Figure 1D). In other words, the same pattern as for the *XBP1u* gene expression was found (Figure 1D).

The chaperone GRP78 (78 kDa glucose-related protein, also called BiP) is considered the initial sensor of an accumulation of proteins in ER with incorrect folding [37]. Under normal conditions, the three ER transmembrane signal transducers (PERK, IRE, and ATF6) remain inactive, anchored to the membrane through its binding to GRP78. However, under conditions of ER-stress, GRP78 dissociates from these proteins leading to their activation [37]. The activation of the signaling cascade of the UPR pathway often leads to a concomitant increase in the expression of *GRP78*, as an autoregulation of the ER-stress situation [16,38]. As shown in Figure 1D, a clear increase in the expression of this chaperone was observed in the pregnant rats supplemented with fructose, becoming statistically significant with respect to glucose-fed mothers.

### 3.2. Ingestion of A 10% w/v Fructose Solution throughout Gestation Increases the Phosphorylation of IRE in Fetal Liver

**Figure 2:**
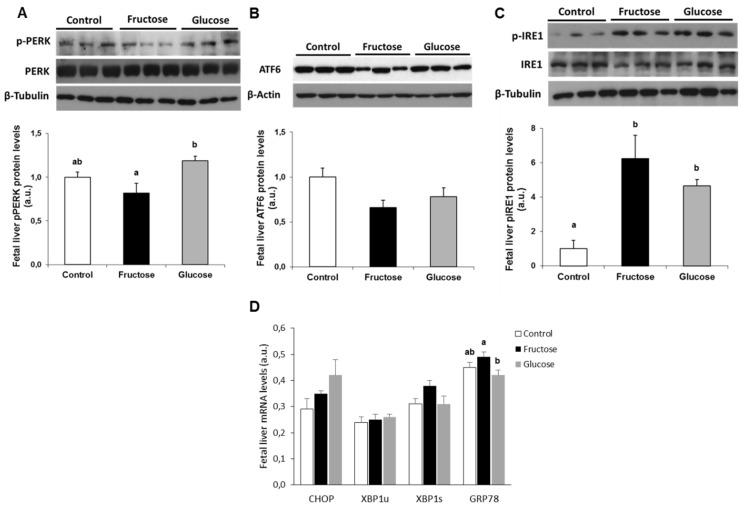
Maternal ingestion of a 10% w/v fructose solution throughout gestation affects ER-stress in fetal liver. Bar plots showing (**A**) the ratio between Thr-phosphorylated form, and the total PERK, (**B**) total ATF6, and (**C**) the ratio between Ser-phosphorylated form, and the total IRE1 proteins in fetal hepatic samples of control (empty bar), fructose- (black bar), and glucose-fed (grey bar) pregnant rats. Values were normalized to β-actin or β-tubulin levels and expressed in arbitrary units (a.u.). (**D**) Bar plots showing the relative hepatic levels of specific mRNA for target genes controlled by the three branches of the unfolded protein response (UPR) from fetuses of control, fructose, and glucose pregnant rats. Results are the mean ± S.E. of values from 6–7 fetal liver pools of the same litter for mRNA gene expression and 5 samples for protein expression. Values not sharing a common letter are significantly different at *P* < 0.05.

In fetal liver, the levels of the phosphorylated form of PERK protein were decreased in the fetuses from mothers supplemented with fructose, being significantly different compared to the fetuses from glucose-fed mothers, in which they were increased (Figure 2A). In accordance to that, the mRNA expression of *CHOP* was augmented, although not significantly, in fetuses from glucose-fed dams (Figure 2D).

Regarding the ATF6-dependent pathway, a slight decline in the nuclear levels of this protein was observed in fetuses from mothers who had taken some type of carbohydrate during pregnancy (although it did not become significant) (Figure 2B). In fact, and according to this result, there were no differences in the levels of *XBP1u* mRNA between the three experimental groups (Figure 2D).

In contrast to the results observed in the other two UPR pathways, an increase in the levels of phosphorylation of the IRE protein was found in the liver of the fetuses from those mothers who had received some type of carbohydrate in the drinking water during gestation (Figure 2C). However, an increase in the levels of *XBP1s* mRNA was observed only in the fetuses from mothers supplemented with fructose (Figure 2D), although it was not significant. This result indicates a slight increase in the splicing of *XBP1u* mRNA to produce *XBP1s* mRNA on the fructose group, as indicated by the ratio *XBP1s/XBP1u* (1.25 ± 0.01; 1.45 ± 0.10; 1.18 ± 0.06 for fetuses from control, fructose-, and glucose-fed mothers, respectively).

As shown in Figure 2D, an increase in mRNA gene expression of *GRP78* was found in liver of the fetuses from fructose-fed mothers, becoming statistically significant with respect to the fetuses from glucose-fed mothers.

### 3.3. Ingestion of A 10% w/v Fructose Solution throughout Gestation Partially Increases ER-Stress in the Placenta

**Figure 3:**
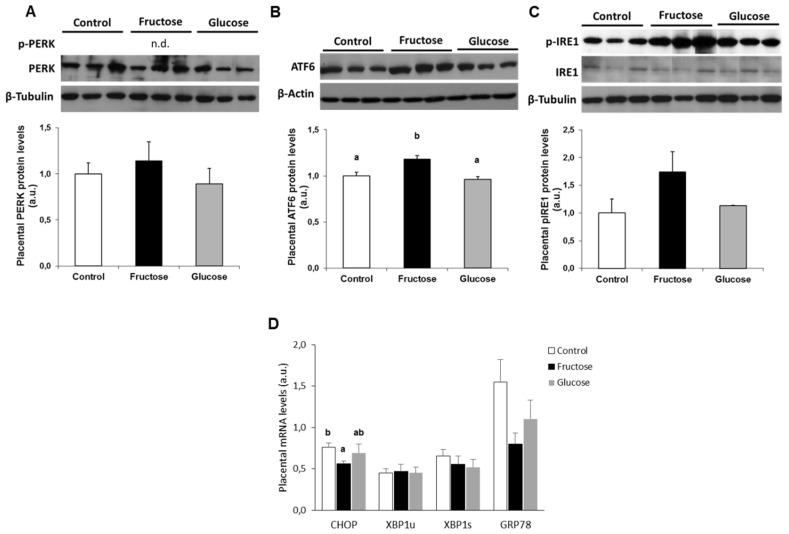
Maternal ingestion of a 10% w/v fructose solution throughout the gestation affects ER stress in placenta. Bar plots showing (**A**) total PERK, (**B**) total ATF6, and (**C**) the ratio between Ser-phosphorylated form, and the total IRE1 proteins in placental samples of control (empty bar), fructose- (black bar), and glucose-fed (grey bar) pregnant rats. Values were normalized to β-actin or β-tubulin levels and expressed in arbitrary units (a.u.). (**D**) Bar plots showing the relative hepatic levels of specific mRNA for target genes controlled by the three branches of the unfolded protein response (UPR) from placentas of control, fructose, and glucose pregnant rats. Results are the mean ± S.E. of values from 6–7 placental pools of the same litter for mRNA gene expression and 5 samples for protein expression. Values not sharing a common letter are significantly different at *P* < 0.05. n.d.: not detectable.

Although PERK protein was observed in placenta (Figure 3A), unfortunately, PERK phosphorylation was not detectable. In spite of this, the expression of one of its downstream effectors (*CHOP*) could be measured. As shown in Figure 3D, *CHOP* mRNA levels were decreased in the placentas of the fructose-fed mothers.

Regarding the nuclear content of ATF6 protein in placenta, in contrast to the results observed in maternal and fetal liver (Figure 1B and Figure 2B), an increase in the amount of ATF6 protein was observed in placenta of fructose-fed mothers, being statistically significant in comparison to the control and glucose-fed groups (Figure 3B). However, this increase in the levels of ATF6 protein was not reflected in the *XBP1u* mRNA levels, as shown in Figure 3D, since they did not differ between the three experimental groups.

Concerning the UPR pathway controlled by IRE, the levels of the phosphorylated form (p-IRE) (Figure 3C) in placenta, as in maternal liver (Figure 1C), showed an increase in fructose-fed mothers versus the other two experimental groups, although in this case it did not become significantly different. Regarding the mRNA expression of *XBP1s*, as shown in Figure 3D, no differences between the three experimental groups were obtained.

Surprisingly, the level of mRNA gene expression of *GRP78* did not display any differences between the three experimental groups (Figure 3D), but rather a trend to diminish in placenta of mothers which received some type of carbohydrate

### 3.4. Fructose Increases XBP1s Protein Levels and Decreases its Translocation to the Nucleus. A Possible Role of Methylglyoxal

**Figure 4:**
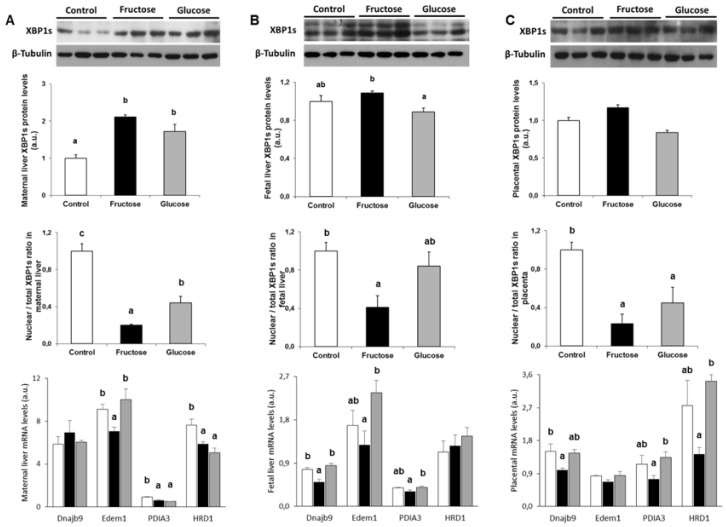
Maternal intake of a 10% w/v fructose solution throughout gestation diminishes nuclear translocation of XBP1s. Western blots and bar plots showing total XBP1s protein (upper panel); bar plots showing the ratio between nuclear XBP1s and the total XBP1s protein (middle panel); and mRNA for XBP1s target genes (lower panel) levels from (**A**) maternal liver, (**B**) fetal liver, and (**C**) placenta of control (empty bar), fructose- (black bar), and glucose-fed (grey bar) pregnant rats. Values were normalized to β-actin or β-tubulin levels and expressed in arbitrary units (a.u.). Bars represent the mean ± S.E. of values obtained from five maternal livers, fetal liver pools, or placenta pools of the same litter for protein gene expression and 6–7 samples for mRNA expression. Values not sharing a common letter are significantly different at *P* < 0.05. XBP1s: spliced X-box-binding protein; Dnajb9: DNA J heat shock protein family member B9; Edem1: endoplasmic reticulum degradation enhancing alpha-mannosidase like protein 1; PDIA3: protein disulphide isomerase 3; HRD1: HMG-CoA reductase degradation protein 1.

Given the heterogeneity of the results found here in the three routes of the UPR in response to maternal intake of liquid carbohydrate, the levels of XBP1s, one of the most representative proteins of ER stress [17], were determined. Thus, the levels of the XBP1s protein were augmented in maternal liver in the two groups supplemented with carbohydrates, being statistically significant with respect to the control group (Figure 4A, upper panel). Moreover, the XBP1s protein levels were increased in the fetuses of fructose-fed mothers, being statistically significant with respect to the glucose group (Figure 4B, upper panel). Similarly, an increasing trend in the levels of XBP1s protein was found in the placenta of the fructose-fed mothers (Figure 4C, upper panel) (that it approached to be significant versus the glucose group, *P* = 0.086), in consonance with the levels found for pIRE (Figure 3C).

To perform its transcriptional activity, XBP1s must migrate to the nucleus and regulate the gene expression of a set of chaperones involved in maturation, folding, and degradation of proteins from the ER [39]. As above mentioned, maternal fructose intake induced the protein expression (Figure 4A–C, upper panel) of the marker of ER-stress XBP1s. However, these results were obtained by measuring the total protein. Therefore, in order to study the degree of translocation to the nucleus of XBP1s, that is, how much of the synthesized XBP1s protein was effectively arriving to the nucleus, the ratio between the levels of the XBP1s protein found in nuclear extract and those found as total protein was calculated.

Surprisingly, in liver and placenta of mothers which ingested some type of carbohydrate (in the form of sugary water) a clear decrease in the ratio of nuclear XBP1s/total XBP1s was found, this being more pronounced in maternal liver of fructose-fed group (Figure 4A,C, middle panel). Furthermore, it was in the liver of the fetuses from fructose-fed mothers where a decrease in this ratio was specifically found (Figure 4B, middle panel). The difference found in fetuses from fructose-fed mothers was significant versus fetuses from control group and marginally different (*P* = 0.064) in comparison to those from the glucose-supplemented pregnant rats. These results reflect a fructose-induced defect in the XBP1s translocation to the nucleus.

These findings found for the nuclear translocation of XBP1s could be influencing its capacity to regulate the gene expression. In order to demonstrate this, several XBP1s target genes related to the endoplasmic reticulum-associated protein degradation (ERAD) system, such as: *Dnajb9* (DNA J heat shock protein family member B9), *Edem1* (endoplasmic reticulum degradation enhancing alpha-mannosidase like protein 1), *PDIA3* (protein disulfide isomerase family A member 3), and *HRD1* (HMG-CoA reductase degradation protein 1) were determined [40,41]. As shown in Figure 4A (lower panel), *Dnajb9* mRNA gene expression was not affected, whereas the levels of mRNA for *Edem1* were specific and significantly decreased in the liver of fructose-fed mothers, and *PDIA3* and *HRD1* gene expression was reduced in the liver of carbohydrate-fed mothers. Regarding fetal liver, as shown in Figure 4B (lower panel), a similar profile to the one observed for nuclear translocation of XBP1s (middle panel) was found for the levels of mRNA of its target genes (*Dnajb9*, *Edem1*, and *PDIA3*), confirming a lower presence (and therefore, a lower transcriptional activity) of the XBP1s in the nucleus of fetal liver from fructose-fed pregnant rats. Finally, concerning the placenta, although nuclear XBP1s translocation was decreased in carbohydrate-fed rats, Figure 4C (lower panel) shows lower levels of mRNA for *Edem1* and, mostly, for *Dnajb9*, *PDIA3*, and *HRD1* in the placenta from fructose-fed mothers.

**Figure 5:**
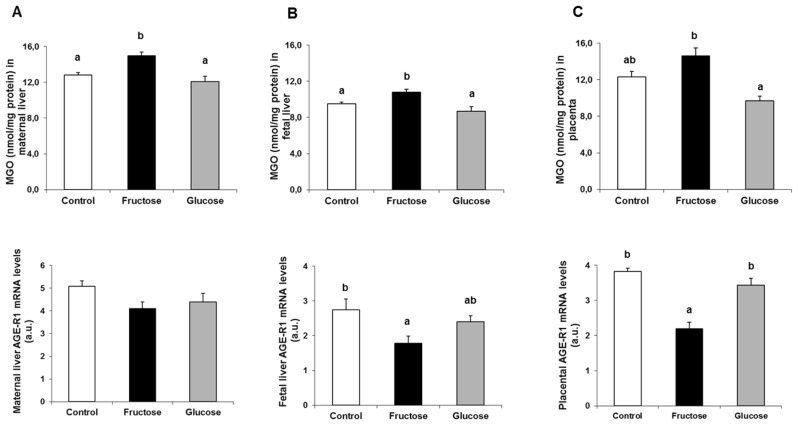
Maternal intake of a 10% w/v fructose solution throughout gestation increases methylglyoxal content in tissues. Bar plots showing the methylglyoxal content (upper panel) and mRNA levels for *AGE-R1* gene expression (lower panel) from (**A**) maternal liver, (**B**) fetal liver, and (**C**) placenta of control (empty bar), fructose- (black bar), and glucose-fed (grey bar) pregnant rats. Bars represent the mean ± S.E. of values obtained from 6–7 maternal livers, fetal liver pools, or placenta pools of the same litter for MGO determination and mRNA expression. Values not sharing a common letter are significantly different at *P* < 0.05. MGO: methylglyoxal; AGE-R1: advanced glycation end products (AGE) receptor 1.

Related to these findings, it has been shown that XBP1s needs to form a complex with proteins such as PI3K (Phosphoinositide 3-kinase) and/or BRD7, to be translocated to the nucleus [41,42,43]. Moreover, methylglyoxal, an important precursor of advanced glycation endproducts (AGE), might be involved in certain changes that are dependent of PI3K signaling [44,45]. Therefore, in order to determine if methylglyoxal levels could help to explain the changes observed in the translocation of XBP1s to the nucleus, its concentration in liver and placenta was determined. As shown in Figure 5 (upper panels), MGO levels displayed practically identical profiles in the three tissues analyzed. A statistically significant increase in livers of mothers and fetuses of the fructose-fed group with respect to the other two experimental groups was observed (Figure 5A,B, upper panels). In placenta, this increase found in fructose-fed mothers was significantly different versus the glucose group, and almost significant (*P* = 0.078) in comparison to the control mothers (Figure 5C, upper panel).

Given that methylglyoxal is a major precursor of AGE, the levels of mRNA gene expression of *AGER1* (AGE receptor type 1), receptor responsible for the elimination of AGEs from the medium [46], were also analyzed. As shown in Figure 5, a significant decrease in the fructose-fed group with respect to the other two groups was observed both in liver of fetuses and placenta (Figure 5B,C, lower panels) and (marginally) significant in liver of pregnant rats (fructose-fed versus control mothers, *P* = 0.079) (Figure 5A, lower panel).

## 4. Discussion

Based on the results obtained, fructose consumption during pregnancy produces ER-stress in the liver of pregnant rats. In fact, a clear activation of two of the three UPR pathways, p-IRE and p-PERK, along with an increase of *GRP78* mRNA levels and a rise in the levels of total XBP1s protein, were found. Interestingly, these changes were not observed in glucose-fed rats. Clayton et al. also found a significant increase in the levels of *GRP78* mRNA in pregnant rats, without any change in *XBP1s* [27]. On the other hand, the decreasing trend of the ATF6 protein gene expression in fructose-fed mothers found in our study is not an unusual case. In fact, other authors have reported either a similar trend in male mice [26], or no changes in female rats [19] subjected to a fructose intake.

On the other hand, an increase in the levels of phosphorylation of IRE in fetuses from fructose-fed mothers paralleled an elevation in the XBP1s expression (both at mRNA levels and total protein). Although the PERK and ATF6 pathways were not affected, all these findings, together with the increase observed in the *GRP78* gene expression, indicate that maternal fructose intake actually increases the hepatic ER stress in their fetuses. Clayton et al. only found an increase in the proportion of *XBP1s/XBP1total* at postnatal day 10, without any effect on fetuses at day 21 of gestation and with no changes in *GRP78* in the two life-times studied [27].

This atypical activation found in the livers of fructose-fed mothers and their fetuses, which does not affect ER-stress in all three UPR pathways, has been previously observed [19,26]. There are more examples, although not related to the consumption of fructose, where a partially activated UPR by ER-stress has been observed [41,47]. These authors suggested that this situation might be advantageous since the cell is able to handle ER-stress without activating the three branches of the UPR so that, for example, it could induce the generation of chaperones, without completely blocking the protein synthesis [41,48].

Similar findings to those found in liver were observed in the placenta, since the consumption of 10% fructose by the mother activated two of the three UPR routes in the placenta, whereas glucose intake did not. Thus, an increase in the levels of both ATF6, p-IRE, and XBP1s proteins was found in placentas from fructose-fed mothers versus the other two groups. To our knowledge, the effect of fructose during gestation on ER-stress of the placenta had not been previously studied, although placental ER-stress is a key factor which may compromise its physiology and functionality. An increase in misfolded proteins might affect the appropriate development and, as a consequence, the placental functioning, including a reduction in cell proliferation, fetal growth restriction, and the activation of pro-inflammatory pathways. However, the expression of various pro-inflammatory genes (*IL1β* and *TNFα*) in placenta in our study showed no differences among the three experimental groups (data not shown).

One of the most striking findings of this study is the fact that the consumption of a 10% fructose solution during gestation caused a decrease in the translocation to the nucleus of XBP1s in liver of pregnant rats. A similar trend was found in glucose-fed rats although not in such an obvious manner (around 20% and 45% for fructose- and glucose-fed, respectively, versus control groups). To our understanding this is the first time a lower translocation of XBP1s to the nucleus has been related to carbohydrate consumption. Curiously, similar results have been reported in animal models of obesity. Thus, Park et al. showed that obese mice developed a severe UPR caused by the inability to activate the XBP1s-dependent machinery of ER-stress regulation, because of a lower translocation of XBP1s to the nucleus [41,42].

This minor translocation of XBP1s to the nucleus detected in the liver of carbohydrate-fed mothers was concomitant with a reduced positive transcription regulation of several chaperones involved in maturation, folding, and degradation of proteins (*Edem1, PDIA3, and HRD1*) [49]. Moreover, XBP1s has been involved in the transcriptional regulation of lipogenesis genes [18]. Although traditionally XBP1s has been shown to increase the transcription of lipogenic genes, such as *SCD1, FAS*, or *ACC*, recent research has established a new facet of XBP1s as an antilipogenic protein [42,50], even in mice subjected to a fructose-rich diet. These studies have proposed that XBP1s is an antilipogenic protein and, therefore, an increased XBP1s activity would reduce the hepatic triglyceride content [50]. XBP1s positively regulates the transcription of genes of chaperones by binding to the respective gene promoters. However, it seems that XBP1s would avoid the activation of the transcription of lipogenic genes through the interaction with some other transcription factor [50]. Thus, a lower presence of XBP1s in the nucleus would reduce its capability to block the transcription of lipogenic genes (*FAS* and *ACC*), increasing hepatic triglyceride content, in consonance with our previous findings in this experimental model [28].

Interestingly, fructose intake during pregnancy produces a diminution in XBP1s translocation to the nucleus in fetal liver, which was not observed after maternal consumption of glucose. Moreover, its transcriptional activity regulating the typical proteins which serve to counteract ER-stress (chaperones and proteins of the ERAD system), was not producing. On the other hand, as mentioned above, XBP1s has recently been established as an antilipogenic protein [50]. In this sense, in fetuses from fructose-fed mothers, a lower XBP1s in the nucleus would diminish the blockade of lipogenic gene (*SCD1* and *SREBP1c*) transcription, which would help to explain previously reported hepatic steatosis in these fetuses [28].

Next, we studied the possible mechanism by which fructose and glucose produced a decrease in XBP1s translocation. Many proteins exist that might be involved (PI3K, BRD7, FOXO1, p38 MAPK) in the interaction with XBP1s and its migration to the nucleus [42,43,51,52]. Curiously, methylglyoxal has been shown to be one of the factors that can influence PI3K protein [44,45]. Interestingly, increased concentrations of MGO were found in the livers of the fructose-fed mothers. That could be altering PI3K and, accordingly, leading to a lower translocation of XBP1s to the nucleus. On the other hand, changes in the hepatic content of MGO were not observed in the glucose group, although XBP1s translocation was also reduced compared to the controls. However, we have previously reported that fructose- and glucose-fed mothers showed increased levels of protein carbonylation (which is considered as AGE) [29]. And, it has been described that protein carbonylation (and other changes in proteins due to modification mediated by AGE) can interfere with many protein functions [53].

Importantly, a reduction in XBP1s translocation to the nucleus also correlated with a higher content of MGO levels in the liver of the fetuses and placentas from fructose-fed mothers, which could indicate that XBP1s nuclear translocation would actually be affected by methylglyoxal. Moreover, levels of protein carbonyls were also increased in the liver of the fetuses and placentas of the fructose-fed mothers [29], in part because of a diminished removal of AGE by a lower presence of AGER1.

Nevertheless, our study has some limitations that could be addressed in the future, apart from difficulties in extrapolating results from experimental animals to humans. These are: (1) The effects observed in the mothers (liver and placenta) are probably due to a direct action of the mother´s sugar (fructose or glucose) intake, however, we do not know whether the effects found in the fetus are due to a direct effect of fructose or the change in the nutritional environment of the mother. Importantly, Vickers et al. using a fructose solution designed to provide 20% of caloric intake from fructose, demonstrated that fructose is able to reach the fetus [54]. (2) We have used simple sugars solutions, and human consumers of sweetened beverages usually consume sucrose (table sugar) or high fructose corn syrup (HFCS). In fact, in our society fructose and glucose are rarely taken separately. Since it has been demonstrated that fructose absorption is improved by the presence of glucose, possibly, the findings observed here would be more pronounced if we had used HFCS instead of fructose or glucose alone.

## 5. Conclusions

Together, these results indicate that despite the fact that ER-stress is induced by maternal fructose intake in the three tissues studied, a severe defect in XBP1s translocation to the nucleus would involve a failure in the resolution of endoplasmic reticulum (ER) stress. In addition, the role of MGO and/or protein carbonylation should not be discarded. Since, we have previously established that maternal fructose induces oxidative stress in fetuses and placentas [29], it could be proposed that fructose-induced oxidative stress is able to modulate UPR (avoiding XBP1s nuclear migration and activation of key ER-stress genes, such as those of the ERAD system), which would lead to unresolved ER-stress and, finally, to chronic ER-stress.
